# Host-*Brucella* interactions and the *Brucella* genome as tools for subunit antigen discovery and immunization against brucellosis

**DOI:** 10.3389/fcimb.2013.00017

**Published:** 2013-05-16

**Authors:** Gabriel Gomez, Leslie G. Adams, Allison Rice-Ficht, Thomas A. Ficht

**Affiliations:** ^1^Department of Veterinary Pathobiology, Texas A&M UniversityCollege Station, TX, USA; ^2^Department of Cellular and Molecular Medicine, Health Science Center, Texas A&M UniversityCollege Station, TX, USA

**Keywords:** intracellular pathogens, vaccines, subunit vaccine, antigen discovery, Brucellosis, reverse vaccinology

## Abstract

Vaccination is the most important approach to counteract infectious diseases. Thus, the development of new and improved vaccines for existing, emerging, and re-emerging diseases is an area of great interest to the scientific community and general public. Traditional approaches to subunit antigen discovery and vaccine development lack consideration for the critical aspects of public safety and activation of relevant protective host immunity. The availability of genomic sequences for pathogenic *Brucella* spp. and their hosts have led to development of systems-wide analytical tools that have provided a better understanding of host and pathogen physiology while also beginning to unravel the intricacies at the host-pathogen interface. Advances in pathogen biology, host immunology, and host-agent interactions have the potential to serve as a platform for the design and implementation of better-targeted antigen discovery approaches. With emphasis on *Brucella* spp., we probe the biological aspects of host and pathogen that merit consideration in the targeted design of subunit antigen discovery and vaccine development.

## Introduction

Infectious diseases account for significant human morbidity and mortality on a worldwide basis. Among these, important subsets of infectious diseases are those caused by intracellular bacterial pathogens. *Brucella*, *Ricketssia*, *Coxiella*, *Chlamydia*, *Burkholderia*, and *Francisella* are important disease causing intracellular etiologic agents. These bacterial agents are included in the CDC select agent list due to their highly infectious nature and their potential use in acts of bioterrorism. Diseases such as salmonellosis, listeriosis and shigellosis are a concern as they represent a threat to food safety. Tuberculosis is a disease caused by a non-select intracellular pathogen but afflicts approximately one third of the human population and accounts for 20% of all human deaths due to infectious diseases (Martin, 2005). The intracellular biology of these agents is the result of extensive and complex interactions with their host that are necessary to establish a niche permissive of pathogen survival and propagation. Despite the risk these diseases represent for public health, there are no licensed vaccines in the United States to counteract many of them. This is a pressing need that is currently being addressed by arduous efforts of the biomedical research community. One of the main focuses of these efforts is to identify new and more effective approaches to discover antigens for immunization formulations and vaccine development. In this review, we describe advances in the field of brucellosis and propose a multistep, antigen discovery approach with the goal of developing vaccines protective against brucellosis and other intracellular pathogen diseases.

*Brucella* spp. classification is based on host preference and virulence (Cloeckaert et al., 2002). Brucellae pathogens have been isolated from a range of species including: *Brucella melitensis* (primarily in goats and sheep), *Brucella abortus* (cattle), *Brucella suis* (swine), *Brucella ovis* (sheep), *Brucella canis* (dogs), *Brucella ceti* (dolphin, porpoise, and whale), *Brucella pinnipedialis* (seal), *Brucella neotomae* (desert wood rat), *Brucella microti* (common vole), and *Brucella inopinata* was recently isolated from human patients (Scholz et al., 2010; Atluri et al., 2011). The reason for host preference is not clear but recent reports characterizing alterations in the genome attempts to explain these species-specific observations (Wattam et al., 2009).

The incidence of human brucellosis is strongly dependent on animal disease prevalence. Brucellosis is a disease that results in great economic loss, particularly in the food animal production sector. Intense regional efforts to control and eradicate animal brucellosis have been successful in some areas but in many parts of the world it continues to be an important disease. As a result, brucellosis is amongst the most common zoonotic diseases with a worldwide annual incidence of 500,000 new cases (Franco et al., 2007). Although humans and animals are susceptible to brucellosis, there is variation in the clinical manifestations in different species.

Brucellae are highly infectious to humans with an estimated dose of only 10–100 organisms being sufficient to establish an infection via the aerogenous route (Bossi et al., 2004). In decreasing order of virulence, *Brucella melitensis*, *Brucella suis*, and *Brucella abortus* are the agents most commonly implicated in human brucellosis (Bossi et al., 2004; Franco et al., 2007; Lucero et al., 2008). Although horizontal human transmission is possible, the most important source of human brucellosis is respiratory or gastro-intestinal exposure to contaminated animal tissue or unpasteurized milk and dairy food products, respectively (Williams, 1970; Eckman, 1975; Taylor and Perdue, 1989; Chomel et al., 1994; Godfroid et al., 2005; Corbel et al., 2006; Mantur et al., 2007). After breaching mucosal barriers, *Brucella* spp. infect submucosal or intraepithelial phagocytic cells and subvert intracellular trafficking pathways. This allows *Brucella* spp. to evade protective mechanisms of host phagocytes to establish an intracellular niche amenable to survival and replication and to provide a means for dissemination. Cell-mediated dissemination to distant organs, especially those of reticulendothelial and reproductive systems, occurs via the circulatory system (Pizarro-Cerda et al., 1998; Adams, 2002; Pappas et al., 2005; Franco et al., 2007). The intracellular nature of *Brucella* spp. favors survival and persistence by evading host immune surveillance. In up to 30% of cases, human brucellosis develops to a chronic disease often accompanied by a combination of undulant fever and myriad non-specific symptoms (Atluri et al., 2011; Skendros et al., 2011). Brucellae has been reported to successfully colonize and cause pathological changes in a variety of organs including those of the central nervous, hemolymphatic, digestive, respiratory, reproductive, musculoskeletal, and cardiovascular systems, an aspect that complicates diagnosis and may result in delay of treatment (Franco et al., 2007; Mantur et al., 2007; Glynn and Lynn, 2008; Seleem et al., 2010; Skendros et al., 2011). Even when diagnosed in a timely manner, the recommended treatment for brucellosis consists of prolonged administration of antibiotics that often fails to completely clear the pathogen (Falagas and Bliziotis, 2006; Seleem et al., 2010).

## Molecular pathogenesis

In order to understand the pathomechanisms of infectious diseases with clinical significance in animals and humans we must first understand the biology of these agents, and their hosts, in order to unravel the interactions that occur at the host-agent interface. Furthermore, understanding of these mechanisms is important for the development of new and effective chemotherapeutic and prophylactic measures. *Brucella* spp. and other intracellular pathogens have acquired mechanisms to evade host innate protective mechanisms to readily gain access to the host intracellular compartment. Once internalized, they evade intracellular protective mechanisms and subsist and multiply free in the cytoplasm or within vacuolar compartments. The organisms within this intracellular niche create a focus of infectious agent that potentially serves as a source for persistent systemic infection and a chronic disease state.

### Internalization

Much effort has been spent elucidating the pathogenesis of *Brucella* spp. Although significant and productive strides have been made since the recognition of human brucellosis in the late 1800's, there is much we do not yet know about the pathogen. However, recent advances in the field have confirmed that the pathogenesis of brucellosis is based on a dynamic and intricate network of interactions that occur at the host-pathogen interface during the invasion, adaptation and replication phases (Rossetti et al., 2011). Deciphering these complex interactions will aid us in elucidating and manipulating the molecular mechanisms responsible for infection and development of disease (Adams, 2002).

Several bacterial and host molecular components that aid or regulate invasion and affect downstream host-pathogen interactions have been identified; however, the full extent of the molecular components and mechanisms acting on either active bacterial penetration or passive uptake of *Brucella* spp. are not fully understood. It is well accepted that brucellae can efficiently breach mucosal epithelial barriers and are capable of infecting several professional phagocytic and non-phagocytic mammalian cells (Murphy et al., 2002; Billard et al., 2005; Hernandez-Castro et al., 2008; Watanabe et al., 2008; Garcia Samartino et al., 2010; Velasquez et al., 2012). Furthermore, the internalization of *Brucella* is a regulated process that involves interactions of surface host and pathogen molecular factors. Accordingly, lipid rafts, complement and Fc receptors are host surface entities for which a role in *Brucella* spp. internalization has thus far been described (Campbell et al., 1994; Moreno and Moriyon, 2002; Watarai et al., 2002; Pei et al., 2008).

**Figure 1:**
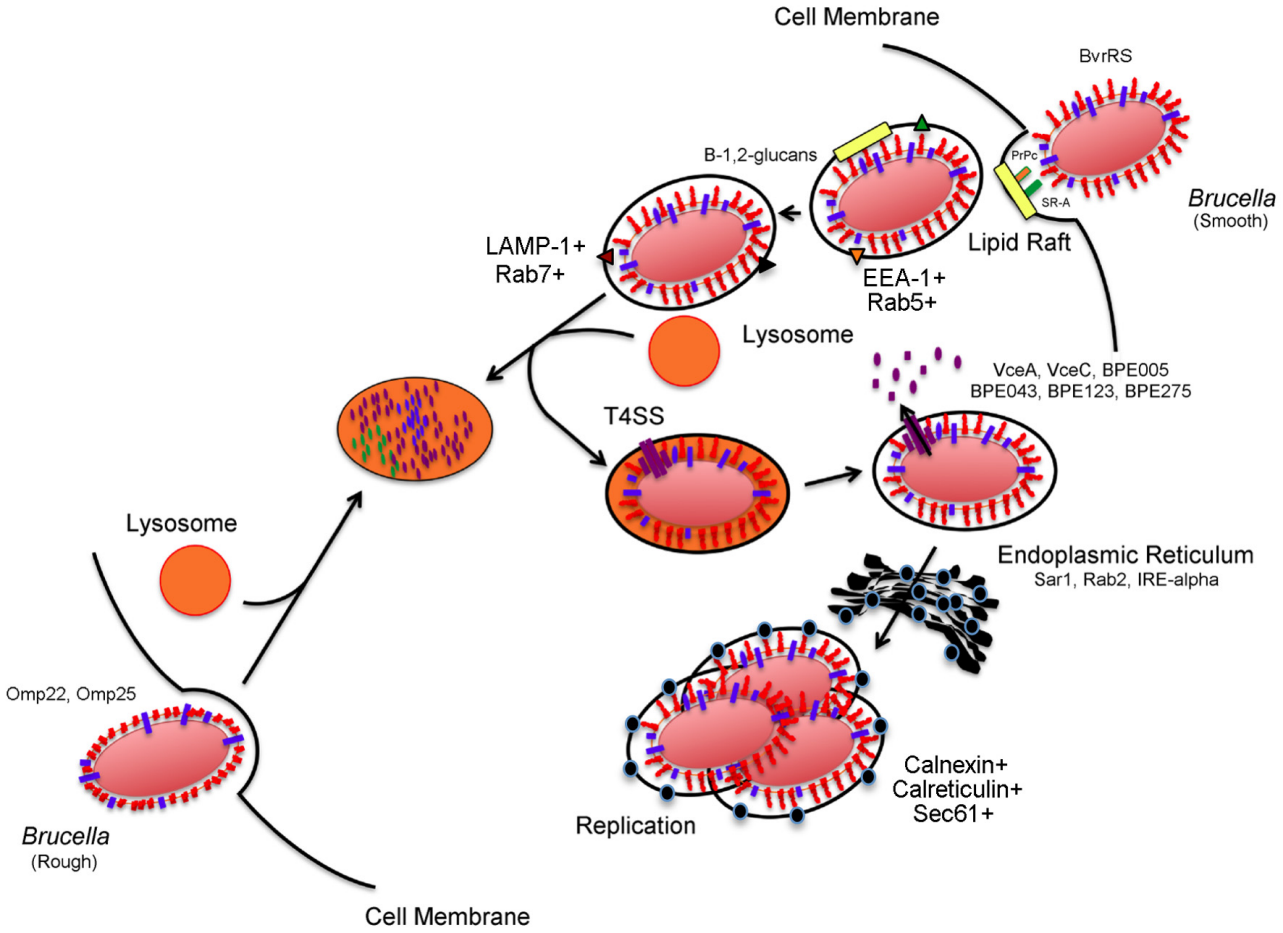
***Brucella* invasion and intracellular trafficking in host mammalian cells.** Smooth *Brucella* is internalized in a vacuole following interactions with host cell lipid rafts, PrPc, and SR-A. *Brucella* derived β-1,2-glucans act by remodeling surface vacuolar lipid-rich domains. The vacuole then fuses transiently with host cell lysosomes for replication- competent bacteria. These transient interactions lead to activation of acid-dependent genes, including type 4 secretion system. Type 4 secretion system substrate proteins are translocated to the cytosol of the host cell where they purportedly act to support trafficking and interactions with the endoplasmic reticulum in order to reach the replicative vacuole. In contrast, internalization and intracellular trafficking of rough *Brucella* is poorly characterized but a role for *Brucella* Omp22 and Omp25 has been demonstrated. The events that occur after internalization are not clear but ultimately lead to lysosomal degradation.

With respect to the pathogen, the outermost component of brucellae is its multifunctional and heterogenous outer membrane (Moriyon and Lopez-Goni, 1998; Jimenez de Bagues et al., 2004; Bos et al., 2007; Martin-Martin et al., 2008; Pasquevich et al., 2009). A major component of the membrane is the LPS, an integral outer membrane molecule with three domains (O-antigen, core oligosaccharide, and lipid A) that has a regulatory role in *Brucella* spp. internalization (Lapaque et al., 2005). Specifically, the O-antigen not only protects the bacterium from intracellular killing mechanisms but also controls its internalization. Brucellae organisms with defects in the LPS (rough) are internalized at a greater rate (i.e., up to 50 fold) than their counterparts with intact LPS (smooth), but are unsuccessful in preventing fusion of the brucellae-containing vacuole with the lysosome (Porte et al., 2003; Jimenez de Bagues et al., 2004; Pei et al., 2008; Haag et al., 2010). These data suggest that cell internalization of *Brucella* spp. is an O-antigen dependent selective process with implications for downstream survival and replication (Figure 1). Additionally, it has been demonstrated that O-antigen is associated with a protective effect on *Brucella* spp. membranes to bactericidal polycations and is necessary for lipid-raft dependent uptake (Moriyon and Lopez-Goni, 1998; Watarai et al., 2002; von Bargen et al., 2012). However, recent reports suggest that additional bacterial factors also present in rough *Brucella* spp. organisms may interact with and mediate lipid raft-dependent uptake (Martin-Martin et al., 2010). These data suggest that while rough organisms may be internalized by lipid raft-dependent and independent mechanisms, smooth brucellae are mostly internalized via lipid raft-dependent mechanisms, perhaps explaining the higher uptake rates observed with rough organisms.

A role in internalization has also been described for brucellae outer membrane proteins. A putative role for outer membrane proteins in internalization is suggested by impaired uptake of *Brucella* spp. with inactivation of the two-component system (TCS), *BvrRS*. The *BvrRS* TCS is a sensory transduction bacterial system that is necessary for full expression of various outer membrane proteins (Sola-Landa et al., 1998; Guzman-Verri et al., 2002; Lamontagne et al., 2007; Viadas et al., 2010). Among those, Omp22 and Omp25 are two membrane proteins for which expression is *BvrRS* dependent. Targeted inactivation of genes *omp22* and *omp25* impaired internalization of rough *B. ovis* but not *B. abortus* (Manterola et al., 2007; Martin-Martin et al., 2008). The basis for these findings is unclear, but suggested explanations range from functional redundancy in the outer membrane to a greater role for LPS in smooth agent internalization and physical obstruction due to LPS-shielding of outer membrane proteins.

Recent reports have identified additional proteins with a role in *Brucella* spp. adhesion and internalization (Castaneda-Roldan et al., 2006; Hernandez-Castro et al., 2008; Martin-Martin et al., 2008; Czibener and Ugalde, 2012; Posadas et al., 2012). The encoding genes or their protein products have homology to adhesin or invasion factors present in other pathogens including *Bartonella bacilliformis* (BMEI0216), *Salmonella enterica* (SP41, BmaC), and enterohemorrhagic *E. coli* (Bab1_2009) (Castaneda-Roldan et al., 2006; Hernandez-Castro et al., 2008; Czibener and Ugalde, 2012; Posadas et al., 2012). Evidence for the involvement of several host and pathogen components in cell internalization of brucellae coupled with poor mechanistic understanding of the process is a reflection of the intricacies and interdependency acting at the host-agent interface.

### Intracellular trafficking and replication

Following internalization, intracellular bacterial pathogens are confronted with a variety of harsh conditions within the host cell and with which they must cope and adapt in order to survive. Their ability to adapt by evading, resisting or subverting intracellular host protective mechanisms is dictated by structural components or elaboration of virulence factors that promote survival, replication, persistence, and systemic dissemination as necessary events to cause disease. Collectively, there are a variety of mechanisms that intracellular pathogens have acquired through evolutionary pressure to establish productive intracellular infections. One of the main intracellular host protective mechanisms is degradation of pathogens within lysosomal compartments. Intracellular pathogens have acquired strategies to avoid the killing mechanisms of host cells by avoiding lysosomal bactericidal contents through escape to the cytoplasm prior to fusion of phagosome with the lysosome (i.e., *Francisella, Shigella, Listeria, Ricketssia*), delay, or arrest of phagosome maturation [i.e., *Mycobacterium* (early endosome), *Salmonella* (late endosome)], modulation of vacuolar intracellular trafficking pathways so as to permit interactions with host organelles critical for the establishment of a replicative niche (i.e., *Legionella*) or resistance to killing by lysosomal contents, as is the case for *C. burnetii* (Starr et al., 2008; Cossart and Roy, 2010; Alix et al., 2011).

Similar to other intracellular pathogens, the complexity of *Brucella* spp. pathogenesis is demonstrated by its ability to resist intracellular killing mechanisms and reach a membrane-bound replicative-competent niche inside host cells (Figure 1). *Brucella* spp. are internalized in a lipid-raft containing vacuole that acquires EEA-1 and Rab5 antigens following interactions with the endocytic pathway (von Bargen et al., 2012). The surface cholesterol-rich lipid rafts are then modified by brucellae-derived β-1,2-glucans (Figure 1) as a necessary step for further maturation of the vacuole (Briones et al., 2001; Roset et al., 2004; von Bargen et al., 2012). As maturation continues, early markers on the *Brucella*-containing vacuole (BCV) are replaced with late endocytic markers LAMP-1 and Rab7 following interactions with late endosomal compartments. These transient interactions between BCV and late endosomes/lysosomes are controlled to allow vacuolar acidification and transcription of acid-dependent bacterial factors (i.e., *vir*B) but prevent vacuolar inclusion of the proteolytic enzyme, cathepsin D (Boschiroli et al., 2002; von Bargen et al., 2012). The *vir*B operon encodes a membrane-associated type IV secretion system (T4SS) used for the secretion of putative bacterial factors (Figure 1) believed to act to modulate the host cell response to support maturation of the BCV (Boschiroli et al., 2002). Although the presence of these effector proteins was only speculative for years, recent research efforts have revealed the identity of several of these T4SS substrate proteins (Figure 1) including VceA, VceC, BPE005, BPE043, BPE123, and BPE275 (de Jong et al., 2008; Marchesini et al., 2011). Nonetheless, the function of these recently identified substrate proteins remains, for the most part, undefined. Ongoing research is focused on the identification of additional effectors and their functions in an effort to understand the precise role that the T4SS and its substrate molecules play in the maturation of the BCV and establishment of the replicative niche (O'Callaghan et al., 1999; Hong et al., 2000; Gorvel and Moreno, 2002; Celli et al., 2003; den Hartigh et al., 2008; Nijskens et al., 2008). After BCV acidification, the vacuole interacts with the ER via Sar1, Rab2, and IRE-α dependent mechanisms (Celli et al., 2005; Qin et al., 2008; Fugier et al., 2009). These interactions lead to BCV acquisition of ER-specific markers that include calreticulin, calnexin, and Sec61 (von Bargen et al., 2012). Furthermore, brucellae replication only occurs after the BCV interacts with the ER (Starr et al., 2012; von Bargen et al., 2012). Although we have a general understanding of the series of events leading up to *Brucella* spp. intracellular replication, the post-replicative events are less clear. Bacterial exit from infected cells is required for intercellular and inter-host propagation (Hybiske and Stephens, 2008). A model consisting of smooth dissociation, intracellular rough replication, bacterial-induced cytotoxicity, and pathogen release has been suggested (Turse et al., 2011). Recently, an egress model where the replicative BCV undergoes further maturation, gains autophagic features and exits as a membrane-bound particle, was reported; however, re-infection of neighboring cells was only demonstrated in human epithelial cells (HeLa) and not the macrophage, the cell type believed to be the major site of *Brucella* spp. replication (David, 2012; Starr et al., 2012).

If the host is permissible to *Brucella* spp. infection then survival, replication and egress results from the ability of the pathogen to concurrently alter the function of multiple host systems. Indeed, active manipulation of host immune response to promote its survival is a fundamental aspect of brucellosis. Evasion of intracellular killing mechanisms affords brucellae the ability to localize within a sub-cellular compartment that favors intracellular replication. Understanding the details and applicable mechanisms of pathogen biology is expected to shed light on the identification of treatment options (Alix et al., 2011). Alterations in intracellular trafficking resulting from host-agent interactions at different stages of the *Brucella* spp. life cycle may be expected to reveal the identity of molecular components or pathways that play an important role in the pathogenesis of the agent (Eriksson et al., 2003; Lucchini et al., 2005; Waddell and Butcher, 2007; Fontan et al., 2008; Wehrly et al., 2009; Fukuto et al., 2010; Rossetti et al., 2011; Pruneau et al., 2012). Most importantly, it is in this intracellular voyage that brucellae actively produce and secrete known and putative molecular effectors that are presumed to aid in the establishment of a replicative niche (Figure 1). Previous studies reveal that known virulence factors such as the *virB* operon, *vjbR*, and Omp25 are upregulated in the intracellular stage of infection (Boschiroli et al., 2002; Wang et al., 2009). Global transcriptional analysis of the intracellular pathogen *Francisella tularensis* in mouse macrophages revealed that greater than 25% of the genes over-expressed in the intracellular phase had been previously demonstrated to have a role in replication or virulence of the pathogen (Wehrly et al., 2009). This finding is relevant to vaccine antigen discovery and vaccine development based on the assumption that virulence factors critical to the establishment of infection are less likely to mutate, their expression during infection is necessary, and, hence, may represent good vaccine candidates.

## Host protective immuno-mechanisms

### Cell-mediated immunity

Host immunity is divided into innate and adaptive immune responses. Due to the chronic nature of many diseases caused by intracellular pathogens, an effective adaptive response is necessary to control disease. Although several components of the immune system contribute to protection against intracellular pathogens, a cell-mediated immune response has been demonstrated to be critical for protection against *Brucella* and other intracellular pathogens such as *Chlamydia, Francisella*, and *Mycobacterium* (Kamath et al., 2009; Shannon and Heinzen, 2009; Karunakaran et al., 2010; Plotkin, 2010). Macrophages and T-cells play crucial roles in protection. Helper T-cell mediated protection is primarily associated with a Th1 T-cell response and persistence (i.e., chronic brucellosis) with a Th2 response (Golding et al., 2001; Giambartolomei et al., 2002; Yingst and Hoover, 2003; Rafiei et al., 2006; Skendros et al., 2007, 2011; Titball, 2008; Perkins et al., 2010). More specifically, studies have demonstrated protective contributions for IFN-γ, IL-12, and TNF-α against brucellosis (Zhan and Cheers, 1995; Zhan et al., 1996; Murphy et al., 2001; Baldwin and Parent, 2002; Brandao et al., 2012). In addition to the Th1 response, CD8^+^ T cells also contribute to protection as mice deficient in MHCI presentation fail to control *Brucella* spp. infection (Oliveira and Splitter, 1995). Protection elicited by passive transfer of CD4^+^ and CD8^+^ T cell subsets corroborate these findings and highlight the importance of T cell cytotoxicity and T-cell driven cytokine-mediated orchestration of the immune response in protection against brucellosis (Araya et al., 1989). The function of dendritic cells in innate and adaptive immunity and their presence at mucosal surfaces makes their study in brucellosis important (Iwasaki, 2007). Dendritic cells have been demonstrated to be permissive of brucellae infection and replication (Billard et al., 2005; Bosio and Dow, 2005). The role of dendritic cells in protection against brucellosis is not completely understood; however, brucellae have been demonstrated to regulate the response of dendritic cells as detailed below (Billard et al., 2005; Iwasaki, 2007). The γδ T-cells may also have a role in the control of brucellosis; however, the precise mechanism for this protection is undefined (Bessoles et al., 2011; Skyberg et al., 2011). Lastly, natural killer cells have cell cytotoxic capabilities and are able to secrete IFN-γ, but a direct role for this cell type in the control of acute brucellosis is not clear (Fernandes et al., 1995; Gao et al., 2011; Vivier et al., 2011). Collectively, these data support a pivotal role for T cell-mediated immunity in protection against brucellosis. Also, these findings support the assumption that T-cell immunity is the single most important response mediating protection from brucellosis (Araya et al., 1989; Elzer et al., 1994; Hoffmann and Houle, 1995; Ko and Splitter, 2003).

### Humoral immunity

The contributory role of antibody to protection against brucellae is less defined than the role of T-cell immunity; however, precise protective mechanisms of humoral immunity against intracellular pathogens may rely on a focused combination of factors that include antibody isotype and function. Protection studies with animal models deficient in B-cell function indicate that this cell type is not necessary for protection against primary infection, yet passive transfer of antibodies from immunized or exposed animals confer protection on naïve animals against brucellosis (Araya et al., 1989; Casadevall and Pirofski, 2006; Goenka et al., 2011). The results suggest that while antibodies have a protective role against re-infection with *Brucella* spp., similar to *Francisella* spp. and *Listeria* spp., their role in protection against primary infection is less explicit (Casadevall and Pirofski, 2006). These data further imply that innate or alternate immuno-protective mechanisms that precede development of humoral immunity are sufficient to control primary infection and the synergistic and/or inhibitory contributions of specific antibodies need to be further explored (Araya et al., 1989; Casadevall and Pirofski, 2006; Titball, 2008; Shannon and Heinzen, 2009; Goenka et al., 2011). While antibodies may be protective against secondary exposure, this protective effect is not always apparent in passive immunity transfer studies suggesting a synergistic, rather than an absolute, protective role for humoral immunity. The basis of these findings may aid in understanding antibody immuno-protective mechanisms. This knowledge will aid in understanding the specific mechanisms that are relevant to protection against *Brucella* spp. and shed light onto the pathogen-specific design and development of therapeutic and prophylactic measures.

### Immunopathology

Intracellular pathogens have strategies or contain structural components to evade and remain undetected by the host immune system. Under evolutionary pressure, *Brucella* spp. have developed elaborate and sophisticated survival-promoting mechanisms *en route* to its establishment as an important pathogen of mammals. These mechanisms endow brucellae with the ability to complete its life cycle in a protected, intracellular, vacuolar compartment. This is evidenced by its capability to manipulate and evade innate and adaptive immunity and other host protective mechanisms through structural component and secreted soluble factor functionality (Barquero-Calvo et al., 2009). Among these, the TcpB molecule is a multifunctional soluble *Brucella*-derived factor that alters cytoskeleton function, inhibits dendritic cell maturation and disrupts the MyD88 signaling pathway (Salcedo et al., 2008; Radhakrishnan et al., 2009, 2011; Sengupta et al., 2010; Chaudhary et al., 2012). A recent report also attributes TcpB an inhibitory function on cytotoxic T-cells during chronic brucellosis (Durward et al., 2012). Another important molecule which effects the immune system is PrpA. PrpA is a *Brucella*-derived B-cell mitogen that supports B cell secretion of the immuno-suppressive cytokine, IL-10 (Spera et al., 2006). This study is in line with the recent finding that B-cells promote brucellae colonization in the mouse (Goenka et al., 2011). The LPS is also a multifunctional, *Brucella*-derived factor with immuno-regulatory capabilities. Specifically, *Brucella* spp. LPS has weak endotoxin (immunostimulatory) properties, protects from complement, and controls antigen presentation through formation of persistent LPS-MHC II complexes (Lapaque et al., 2006; Barrionuevo et al., 2008). Several outer membrane proteins have also been demonstrated to support *Brucella* spp. infection and survival. Brucellae Omp25 regulates TNF-α secretion and prevents apoptosis of macrophages (Gross et al., 2000; Jubier-Maurin et al., 2001; Billard et al., 2007). Anti-apoptotic properties have been demonstrated for the Omp2b porin in macrophages and yeast (He et al., 2006; Laloux et al., 2010). Interestingly, recent reports describe apoptosis of *B. abortus* infected T-lymphocytes and astrocytes (Garcia Samartino et al., 2010; Velasquez et al., 2012). The basis for this selectivity is not clear but the inherent cellular role in promoting brucellosis may be important.

## Immunization approaches

Vaccination is the most efficient and inexpensive method to counteract infectious diseases (Oyewumi et al., 2010). Historically, vaccinology has relied on immunization with live-attenuated organisms or subunit antigens as two of the main approaches to elicit host immunoprotection against infectious agents. However, the success of the immunization approach used against any given pathogen is influenced by multiple factors including pathogen biology, safety, and acceptable levels of protection.

### Live attenuated vaccination

Although not always the best option from a biosafety standpoint, live-attenuated vaccination to combat disease caused by intracellular infectious agents is the approach that, in most cases, confers the highest degree and longest duration of protection (de Veer and Meeusen, 2011; Levitz and Golenbock, 2012). The superior protective efficacy of live attenuated vaccines against intracellular pathogens relies in part on their ability to elicit robust cell-mediated immune responses (Seder and Hill, 2000; Titball, 2008). Accordingly, protective vaccines exist for *Brucella* spp. and other intracellular pathogens but aspects such as residual virulence, hypersensitivity, and lack of protective efficacy across age groups makes them unfit for immunization of humans and, therefore, many have failed to meet approval standards for human use in the United States (Blasco and Diaz, 1993; Schurig et al., 2002; Waag et al., 2002; Ashford et al., 2004; Martin, 2005; Wallach et al., 2008; Rockx-Brouwer et al., 2012). Accordingly, intense research efforts have been placed on identifying live-attenuated vaccines directed at brucellae with an acceptable combination of safety and protective properties (Titball, 2008; Ficht et al., 2009). For instance, identification of virulence-related *Brucella* spp. genes identified via high-throughput mutagenesis studies has resulted in several promising vaccine candidates that are currently in various stages of development (Wu et al., 2006; Ficht et al., 2009; Perkins et al., 2010). Even though live-attenuated organisms provide adequate levels and long-lasting protection, there are concerns associated with the production, stability, and administration of such vaccines (Levitz and Golenbock, 2012). Manipulation and production of live attenuated vaccine strains often necessitate stringent bio-containment. Additionally, risk for reversion to virulence exists and differentiation between exposed and immunized subjects can be challenging and complicate immuno-surveillance efforts. Lastly, there is an ever-present risk of disease in both healthy and the ever-growing population of immune-compromised subjects (Frey, 2007; Chen et al., 2010; Oyewumi et al., 2010).

### Subunit vaccination

In efforts to address concerns associated with live-attenuated vaccination, subunit vaccinology is increasingly gaining support as an alternative approach. However, there are many challenges associated with the development of a protective subunit vaccine against *Brucella* spp. One of the major impediments to progress in this field can be attributed to the difficulty involved in the identification of protective antigens. Second, delivery of soluble subunit antigen primarily targets a humoral immune response as opposed to the crucial cell-mediated immunity needed to protect against *Brucella* spp.; however, recent developments in delivery of antigen are designed to address these concerns. Lastly, it is unlikely that protection resulting from subunit vaccination against *Brucella* spp. will depend on a single antigen. An approach to identify antigens that act synergistically to elicit protective immunity must be considered (Titball, 2008; Plotkin, 2010).

Traditional approaches to subunit vaccine development, such as those based on protective efficacy of bacterial fractions with subsequent identification of specific protective antigens, and the use of serology to identify immuno-dominant antigens, has been a common practice (Rappuoli, 2000; Chen et al., 2011). These methods can be tedious and expensive and often give an unsatisfactory outcome. As a result, these approaches have led to the identification of several subunit antigens that elicit marginal or variable levels of protection against brucellosis and which seldom approach the levels of protection elicited by live-attenuated vaccination (Corbel, 1976; Tabatabai and Pugh, 1994; Oliveira and Splitter, 1996; Al-Mariri et al., 2001; Velikovsky et al., 2003; Ashford et al., 2004; Cassataro et al., 2005, 2007; Martin, 2005; Pakzad et al., 2009; Pasquevich et al., 2009). The limited success of this approach and advances in the understanding of host-*Brucella* spp. interactions has triggered the search for more efficient approaches to antigen discovery and vaccine development.

In the development of subunit vaccines, antigen selection and delivery platform are two key components that merit careful consideration (de Veer and Meeusen, 2011; Levitz and Golenbock, 2012). Significant advances in the fields of immunology, host-agent interactions, microbiology, and bioinformatics have provided insight for the strategic design of targeted tools with protective antigen identification capabilities

## Antigen selection

In the wake of emerging and re-emerging infectious diseases and bioterrorist threats, the need for protective vaccines approved for human use is a priority of research on agents such as *Brucella* spp. in the United States. To this end, a clear understanding of the molecular interactions between *Brucella* spp. and the host and the identification of antigenic determinants that stimulate protective host immune responses are fundamental and vital pieces of information that need to be considered. However, the development of a vaccine is further complicated by the peculiarity of *Brucella* spp. in that it lacks many of the classical virulence factors and has specific mechanisms to counteract host protective mechanisms in order to promote its persistence (Atluri et al., 2011).

One important deterrent to the advancement of subunit vaccine development is the historically ineffective manner of antigen discovery. The identification of immunogenic proteins based solely on humoral immunity is a method that is often suggested or experimentally explored, but this approach undermines or does not always correlate with immune responses determined to have clear relevance to protection against *Brucella* spp. and other intracellular pathogens, i.e., T cell immunity (Rappuoli, 2000; Liang et al., 2010; Chen et al., 2011; Zhao et al., 2011). The availability of complete genomic sequences for many brucellae and their hosts has made the study of functional genomics, proteomics, and immunomics in the context of host-brucellae interactions possible.

**Figure 2:**
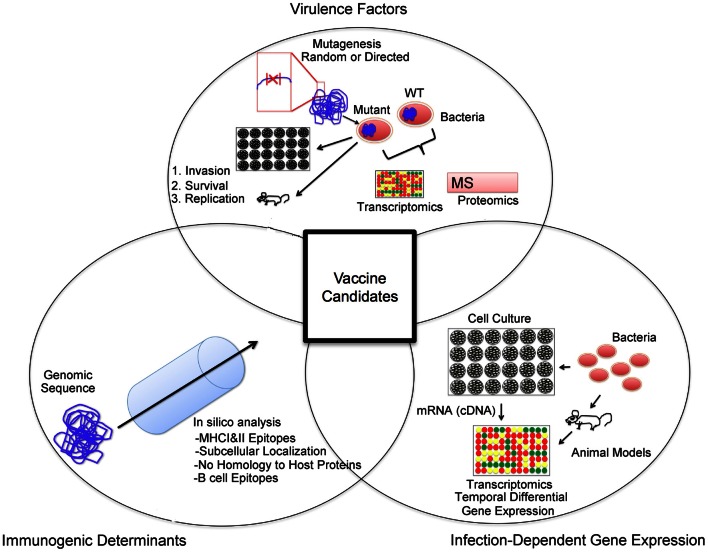
**Vaccine candidate selection approach.** An important aspect of a vaccine candidate is antigenicity. *In silico* analysis of available genomic sequences can aid in the selection of open reading frames that code for desired properties such as T and B cell epitopes, subcellular localization (i.e., outer membrane proteins), and a lack of homology to host proteins. Secondly, antigens with evidence for a role in pathogenesis are often targeted in the identification of vaccine candidates. Identification of factors important for invasion, survival, and replication can be performed via mutagenesis studies in the mouse or cell culture systems. Additionally, comparative transcriptomic and proteomic studies of wild type and mutant pathogen strains can be carried out to identify potential virulence factors. Lastly, the priming of an immune response to a specific antigen relies on its availability. In order to identify antigenic targets present during infection, infection-dependent gene expression studies may reveal suitable targets.

The results obtained from these comprehensive experiments have started to yield data that will increase the understanding of not only the pathogenesis of disease but will also aid in characterizing molecule-specific immunogenicity. Notably, the availability of genomic sequences has led to the development of algorithms for the *in silico* prediction of proteins with desirable structural, (i.e., outer membrane proteins), functional (i.e., adhesin), and antigenic (B cell and T cell epitopes) determinants (Figure 2). Following from these predictions, each protein can be expressed and tested for immunogenic and protective potential, in an approach known as reverse vaccinology (Rappuoli, 2000; Sette and Rappuoli, 2010). Taking into consideration host protective mechanisms, antigen selection via the reverse vaccinology approach can be tailored to identify antigens that contain a combination of antigenic determinants and functional, conserved, and structural properties (He et al., 2010). In its nascent stage, the use of reverse vaccinology has yielded promising results and progress is being made toward the development of vaccines against several pathogens (Sette and Rappuoli, 2010; Bagnoli et al., 2011). Additionally, this approach is often used in an effort to narrow the number of candidate antigens to a working pool for validation and evaluation. Specifically, reverse vaccinology has aided in the identification of antigens that have protective antigenic determinants from various intracellular pathogens including *Chlamydia*, *Ehrlichia, Francisella, Mycobacterium*, and may represent a viable approach to the identification of antigens for immunization against brucellosis (McMurry et al., 2007; Finco et al., 2011; Zvi et al., 2011; Liebenberg et al., 2012; Sundaramurthi et al., 2012).

In addition, availability of genomic sequences has led to the development of whole proteome assays that have been used to determine antigen-specific antibody responses against *Brucella* spp. (Liang et al., 2010). A clear advantage of these assays is that they will aid in overcoming some limitations of traditional assays such as 2D gels, which are based on total bacterial lysates and its signal is dependent on the quantity of antigen in the preparation. While this approach will aid in screening immuno-dominant antigens that have potential vaccinogen value, this approach must be used as an ancillary rather than the sole criterion for antigen selection as humoral immunity does not always correlate with the more protective responses as described above. In addition, knowledge of the precise brucellae-specific protective mechanisms of humoral immunity (i.e., antibody isotype) may aid in further focusing selection on the most relevant targets.

**Figure 3:**
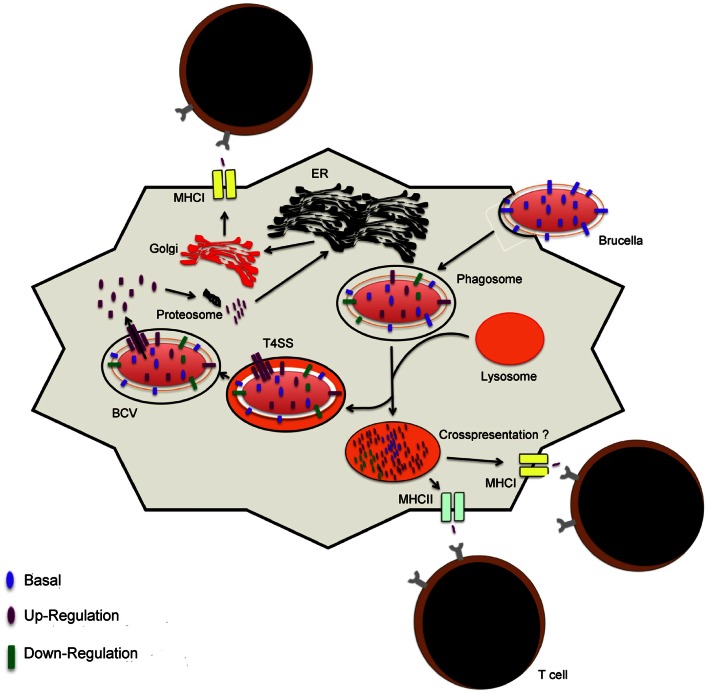
**Model for antigen presentation during *Brucella* infection.** Upon internalization of *Brucella*, the conditions in the vacuole trigger changes in the gene expression profiles. Interactions or fusion with the lysosomes result in changes that support intracellular replication or lead to *Brucella* degradation, respectively. Peptides that result from degradation are presented via MHCII to helper T cells or, presumptively, to cytotoxic T cells via the vacuolar pathway of cross-presentation. Intracellular *Brucella* proteins are processed for presentation via MHCI molecules. Proteins are processed into peptides by host proteosomes in the cytosol. Peptides are loaded into MHCI molecules in the endoplasmic reticulum and packaged in the golgi apparatus for transportation to the surface of the host cell for presentation and activation of T cells.

One other important aspect of infectious diseases that is relevant to vaccination is host-pathogen interactions as these may reveal the dynamics of bacterial antigen quantity and shed light into their putative role in infection (Figure 2). The availability of genomic sequences has led to development of assays, notably microarrays, targeted at the study of host-agent interactions at the gene and protein levels. First of all, suitable pathogen antigen targets must be present in sufficient quantities during infection in order to be recognized by the immune system. As described above, brucellae secrete factors in order to evade intracellular protective mechanisms and establish a niche in which they can replicate. Putatively, these antigens are secreted into the cytoplasm to alter normal function of host cells and manipulate intracellular trafficking (Figures 2, 3). As a result, these factors or those associated with them (i.e., secretion system) can potentially be processed and presented in MHC I molecules via the endogenous pathway (Figure 3), in alternate presentation pathways to CD8^+^ T cells, a T cell subset important for protection against brucellosis (Oliveira and Splitter, 1995; Blanchard and Shastri, 2010; Grillo et al., 2012). Similar to *Mycobacterium* and *Salmonella*, protection studies in *Brucella* have demonstrated that immunization with bacterial factors that are up-regulated during infection are of value in eliciting protective immunity (Rollenhagen et al., 2004; Lowry et al., 2011; Kao et al., 2012). Although work with *Brucella* spp. is limited, evidence for early success merits further investigation. Additionally, these antigens could potentially be virulence factors and serve as better antigens in a vaccine formulation based on the presumption that they are conserved and may be less prone to mutation due to their role in pathogenesis. For instance, LPS and the T4SS are important virulence factors with vital roles in intracellular survival that are present in the pathogenic strains *B. melitensis*, *B. abortus*, and *B. suis* (Carle et al., 2006; Wattam et al., 2009).

Overall, we propose that antigen selection for the development of subunit immunization regimen for *Brucella* spp. must include antigens that: (1) Contain antigenic determinants with protective relevance, a process that may be aided by *in silico* analysis of genomic sequences; (2) Are present at adequate levels during infection in an effort to provide a target for recognition and elimination of infected host cells by the primed immune system; and (3) Have a link to the pathogenesis of the agent with the intent to minimize the risk of mutation and resistance. We propose that consideration of host protective mechanisms and antigenic determinants, *in vivo* expression of pathogen molecular factors, and brucellae virulence-related factors in an antigen selection scheme will lead to the identification of immuno-protective *Brucella* spp. antigens (Figure 2).

## Enhancing protective immune responses of subunit vaccines

Two important considerations in subunit vaccination are adjuvant or delivery vehicle and route of immunization. Adjuvants are compounds co-administered with antigen that have the ability to affect the potency and quality of antigen-specific immune responses. In part due to their ability to stimulate innate immunity, adjuvants influence the ensuing adaptive immune response directed at the antigen (Coffman et al., 2010). Research efforts aimed at deciphering the stimulatory effects of adjuvants have aided in their rational selection for inclusion in subunit vaccine formulations when the general protective immuno-mechanisms against the pathogen of interest are known. Adjuvants almost invariably stimulate humoral immunity but can be selected based on their ability to stimulate a combination of Th1, Th2, Th17, and CD8^+^ T-cell responses (Coffman et al., 2010). From these, targeting activation and proliferation of CD8^+^ T cells and stimulation of a Th1 response are important when considering vaccine development for brucellae. While Poly-IC, imiquimods, CpG, ISCOMS (immuno-stimulatory complexes) have been demonstrated to result in Th1 and CD8^+^ T cells, CAF10 and MPL stimulate Th1 skewed responses (Coffman et al., 2010; Duewell et al., 2011). Despite the role that Th1 and CD8^+^ T cell responses play in protection against brucellosis, the majority of *Brucella* spp. subunit immunization studies have and continue to primarily use Alum (aluminum salts) or Freund's adjuvant (FA) in immunization formulations (Pasquevich et al., 2009, 2011; Lowry et al., 2011; Yang et al., 2011a,b; Fu et al., 2012; Goel and Bhatnagar, 2012; Jain et al., 2012; Li et al., 2012). This practice is inconsistent with the protective mechanisms against brucellosis since Alum is primarily associated with a Th2 response, FA elicits a mixed Th1/Th2 response and both have no demonstrated ability to stimulate of CD8^+^ T cell responses (Coffman et al., 2010); however, the use of Alum may be associated with its acceptance for use in human subjects.

The field of adjuvant/antigen subunit vaccine formulation is an area that merits further exploration in the context of *Brucella* spp. vaccinology. An immunization study revealed that protection against virulent brucellae challenge was dependent on adjuvant where co-administration with CpG ODN, a TLR agonist, but not non-CpG ODN or protein (P39) alone resulted in protection against challenge (Al-Mariri et al., 2001). Another study demonstrated that mice inoculated with L7/L12 in liposomes had a greater ability to clear *Brucella* spp. than mice immunized with antigen and FA (Mallick et al., 2007). Nonetheless, another study reported no difference in protection regardless of whether MPL, FA, or Alum was used (Velikovsky et al., 2003).

The use of recombinant vectors is another viable option for delivering subunit antigens. Viral and bacterial vectors that are safe for use in vaccine formulations have been developed and are currently used for immunization with subunit antigens. One of the appealing characteristics of this type of vaccination platform is their ability to induce cell-mediated immunity, particularly cytotoxic T lymphocytes (Harms et al., 2009; Rollier et al., 2011). Given the role of these cells in protection against intracellular pathogens, this is an appealing approach in brucellosis subunit vaccinology. The use of *E. coli* as the delivery vehicle for recombinant *Brucella* spp. antigens (i.e., P39) demonstrated that this platform induced only marginal levels of protection (Al-Mariri, 2010; Al-Mariri et al., 2012). However, the combination of the recombinant bacterial vector with a TLR agonist enhanced the protective effect of the formulation to a level that approached that elicited by a live-attenuated vaccine strain (Al-Mariri et al., 2012). A point of consideration is that these studies used the non-invasive *E. coli* strain BL-21, which perhaps affected downstream antigen processing as a result of the mode of antigen uptake (i.e., active vs. passive) by antigen presenting cells. The elaboration and use of a recombinant invasive *E. coli* strain to deliver antigens revealed promising results in mouse studies (Harms et al., 2009; Gupta et al., 2012). Lastly, the use of *Lactococcus* and *Ochrobactrum* over-expressing *Brucella* spp. antigens has also been evaluated and shown to confer partial protection against challenge (Saez et al., 2012). However, the fact that these two bacterial strains are avirulent may again have an effect on antigen presentation as they may be highly attenuated intracellularly and potentially result in limited antigen production or be internalized by APCs through routes that do not fully stimulate desired immunity. Other promising bacterial vectors such as *Salmonella*, an intracellular pathogen, are also available but have not been evaluated in the context of brucellosis (Galen et al., 2009). The advantages of using the *Salmonella* live vaccine are that this bacterium is a potent stimulator of innate immunity, targets internalization by antigen presenting cells and has an intracellular lifestyle with a well-defined capacity for protein production and secretion in the intracellular compartment. The use of recombinant viral vectors is another area that may prove useful in delivery of subunit antigens. Despite its widespread use in the literature, its use in brucellosis is limited (Perkins et al., 2010).

Another area that merits consideration in *Brucella* subunit vaccinology is the route of immunization. Immunization with *Brucella* LPS complexed with *Neisseria* OMP resulted in better protection to intranasal challenge following parenteral compared to intranasal immunization (Bhattacharjee et al., 2006). However, potential antigen degradation via the mucosal route was not accounted for in this study. Similarly, lipoproteins co-delivered with cholera toxin conferred protection against *Brucella* challenge in animals immunized via the mucosal route but the degree of protection was not as high as that obtained in mice immunized via the parenteral route (Pasquevich et al., 2011). The identification of measures to enhance mucosal protective immunity is an area of interest in brucellosis vaccinology as the main route of brucellae infection is through mucosal surfaces.

Entrapment of antigen in particles for delivery is yet another option for subunit immunization. In the context of *Brucella* subunit vaccinology little is known about particulate antigen delivery. Several physical aspects of particles have been linked to their effect on the immune system and include particle size, chemical composition, erosion rate, surface chemistry, and immunization route as thoroughly reviewed elsewhere (Oyewumi et al., 2010; Rice-Ficht et al., 2010). Other than affecting size-dependent penetration of anatomical barriers, there is evidence that supports particle size playing a role on the quality of the ensuing immunity relative to humoral and/or cell-mediated responses following immunization (Oyewumi et al., 2010). Additionally, the use of particles may aid in preventing antigen degradation when mucosa is the intended route of immunization, enhance uptake by dendritic cells, and macrophages, and act as a focus for prolonged antigen release acting as a self-boosting mechanism (Oyewumi et al., 2010). In brucellosis research, delivery of antigen in the form of outer membrane vesicles, in liposomes, or in polymeric particles has been explored to a limited extent (Estevan et al., 2006; Munoz et al., 2006; Mallick et al., 2007; Jain-Gupta et al., 2012). Therefore, antigen delivery in polymeric particles is an area with encouraging potential in subunit antigen immunization against brucellosis. Given that the physical properties of particles can have an effect on the resulting immune response, these can potentially be designed to deliver antigen and stimulate antigen-specific protective immunity against brucellosis.

## Conclusions

The antigen, delivery vehicle or adjuvant, and delivery route are key components that merit careful consideration in the development of a subunit vaccine against brucellosis (de Veer and Meeusen, 2011; Levitz and Golenbock, 2012). However, the identification of suitable antigens may represent the most challenging aspect. Significant advances in the fields of immunology, microbiology, and bioinformatics and their application to the understanding of host-agent interactions have provided insight for the strategic design of better-targeted tools that aim to identify protective antigens.

There are important criteria that must be met in an effort to maximize the probability of arriving at the identification of a suitable antigen. First, the candidate immunogen must have antigenic determinants. As discussed above, a T-cell mediated response is generally critical for protection against brucellosis. Therefore, the inclusion of antigens with MHC I and MHC II epitopes for the priming of CD8^+^ and CD4^+^ T cells, respectively, should be included in the screening of candidate antigens. Accordingly, an antigen delivery vehicle that targets both the endogenous and exogenous antigen presentation pathways needs to be considered. In order to target protective humoral immunity, a deeper understanding of the precise mechanisms and associated antibody isotypes, quantities and temporal dynamics involved in the protection against *Brucella* spp. is needed. Secondly, the level of antigen present during infection must be considered for two main reasons. One, high antigen levels during infection may indicate that those antigens are necessary for adaptation or replication of the pathogen. Two, availability of suitable quantities of antigen to serve as a source for antigen processing and presentation may enhance elimination of infected cells as a result of recognition by the immune system. Thirdly, the adjuvant or delivery platform selection is a critical component of vaccine formulation. The delivery system must support the development of a Th1 and CD8^+^ T cell immune responses, consistent with the immuno-mechanisms that confer host protection against brucellae. Finally, appropriate combination of adjuvants and/or delivery systems may be required to potentiate desired immune responses that aid in enhancing protective efficacy. We propose that all these parameters merit consideration in the discovery of *Brucella* spp. subunit antigens and vaccine development.

### Conflict of interest statement

The authors declare that the research was conducted in the absence of any commercial or financial relationships that could be construed as a potential conflict of interest.
